# Social media infodemics and social distancing under the COVID-19 pandemic: public good provisions under uncertainty

**DOI:** 10.1080/16549716.2021.1995958

**Published:** 2021-11-21

**Authors:** Susumu Cato, Takashi Iida, Kenji Ishida, Asei Ito, Hiroto Katsumata, Kenneth Mori McElwain, Masahiro Shoji

**Affiliations:** a Institute of Social Science, University of Tokyo, Tokyo, Japan; b Graduate School of Arts and Sciences, University of Tokyo, Tokyo, Japan

**Keywords:** Social distancing, infodemics, risk aversion, public good, social media

## Abstract

This debate examines the impact of infodemics – an over-abundance of information – on social distancing during the COVID-19 pandemic. Because of its external effects, social distancing behavior (SDB) shares fundamental properties with public goods, whose potential for undersupply has been examined extensively in the social sciences. Although the negative effects of infodemics have been emphasized by governments and international organizations, theoretical models suggest that infodemics may work as a mitigation mechanism. That is, infodemics may enhance people’s SDBs. Based on original survey data, we show that media exposure can positively increase SDB. We conclude by discussing two public health implications. First, the media plays an important role in motivating SDB. Second, even if infodemics can increase SDB, we must be wary of their ability to pose other, non-negligible dangers.

## Background

In public health, social distancing is the practice of staying at home and avoiding out-of-the-home interactions with others in order to reduce the spread of viral infections [1] (see the online Appendix for a difference between social distancing and physical distancing). Under the current COVID-19 pandemic, it shares fundamental properties with *public goods*, because social distancing behavior by one person has a positive external effect on the welfare of others. However, this nature of social distancing engenders a *free-rider problem* [2], under which individual levels of social distancing are likely to be socially suboptimal, at least on average. Because governments cannot completely regulate every citizen’s behavior, voluntary choices to socially distance matters. This is one of the reasons why it has been difficult to control the COVID-19 pandemic.

Notably, the COVID-19 pandemic has been accompanied by *infodemics* [3–5]. According to the World Health Organization (WHO), an infodemic is ‘an over-abundance of information – some accurate and some not – that makes it difficult for people to find trustworthy sources and reliable guidance when they need it’ [6]. In social media, there is a tremendous flow of information about (potentially wrong) protective measures and rumors of government conspiracies. Those who constantly access Twitter, Facebook, or YouTube receive new information every minute, leaving them less time to judge the accuracy of each piece of information. Notably, earlier studies have shown that 16% of tweets with a COVID-related hashtag leads to advertising or non-related issues [7], and that 25% of the Top 12 YouTube videos on COVID-19 include misleading information [8]. International organizations such as the WHO have suggested that infodemics can have a dire negative impact on the COVID-19 pandemic, and they – along with national governments – have sought to correct misinformation about COVID-19. As such, researchers and organizations alike have stressed the importance of eliminating misinformation [2,4].

People’s attitudes toward social distancing vary as a function of demographic characteristics, as well as socio-psychological factors such as trust and altruism [9–11]. Relatedly, one effect of infodemics on individual behavior is the introduction and enhancement of uncertainty about necessary levels of social distancing. Under the COVID-19 pandemic, especially during its early phases, people inevitably face uncertainty about infections, because the virus/disease is novel. As such, they cannot know both the probability of being infected and the magnitude of the illness. Infodemics through social media generate an enormous amount of noise, which is a source of uncertainty, and thus risk and ambiguity due to the pandemic can be magnified.

While the deepening of uncertainty can change an individual’s choice, we argue that this may not necessarily manifest in socially negative ways. Indeed, earlier work notes that uncertainty may enhance people’s social distancing [12], as can social media usage [13]. Our paper links these factors explicitly and argues that high frequencies of social and media access can increase social distancing. Drawing on the literature on public goods and original survey data, this study examines how infodemics can affect voluntary incentives to undertake social distancing behaviors.

## Social distancing as public goods under uncertainty

The canonical model in economics suggests that the provision of public goods is insufficient unless there is legal enforcement or strong policy intervention by the government, because individuals have an incentive to free-ride off the actions of others [14,15]. An individual’s provision of public goods is decreasing in other’s provision: if others’ provision is enough, there is no incentive to provide more, but if no others provide public goods, then each person has a strong incentive to provide some. This observation has been extended to multiple issue domains and tested in various fields of the social sciences [16,17], including in relation to its public-health implications [18,19]. Given this theoretical observation, it is of interest to examine when and how an incentive to provide public goods becomes stronger. The lack of such incentives generates social inefficiency due to the free-rider problem; its presence can, instead, improve efficiency. Throughout this paper, we refer to factors that discourage free-riding as the *mitigation mechanism*.

A simple model of public goods can be extended in various ways to understand the mitigation mechanism. One prominent direction has been to incorporate the existence and effect of uncertainty on individuals’ decision to contribute public goods. Since the magnification of uncertainty is one of the distinguishing effects of infodemics, observations from this theoretical approach offer helpful implications for our understanding of voluntary social distancing behavior.

Two types of uncertainty are distinguishable: *risk* and *ambiguity*. The concept of risk is associated with situations where the probabilities over outcomes (or states) are known. Put plainly, risk is captured by the variance of outcomes, and the risk-aversion of an individual is defined as disutility from larger variances. A key theoretical observation is that risk-averse individuals tend to overcome free-rider incentives and increase their provision of public goods – under certain theoretical conditions – if they expect greater variation, and hence possibility of severe under-provision, of those public goods [20]. This mechanism can apply to the COVID-19 pandemic as follows. Consider an individual who is choosing a degree of social distancing behavior. The risk on public goods corresponds to a lack of information about how other people will follow social distancing measures, or a lack of knowledge on how social distancing behaviors affect one’s infection probability. Even if individuals know the probabilities over the outcomes, they cannot know what outcome will actually result due to the variance associated with the risk. Social media infodemics introduce tremendous noise about correct levels of social distancing, and thus enlarge the variances of outcomes produced by social distancing on infection risk. When there is a risk about others’ social distancing behavior and the resulting infection probability, individuals cannot safely freeride on others’ contributions. Put differently, an individual cannot be sure whether he/she can safely dine out or engage in leisure activities due to the lack of information. Then, if the individual is sufficiently risk-averse, he/she chooses a high degree of social distancing.

The second type of uncertainty, ambiguity, can also reduce free-riding incentives. In the literature on decision theory, ‘ambiguity’ is distinguished from ‘risk’, in that it refers to uncertainty about the probability distribution of future outcomes. Simply put, an individual does not even know the risks. In the case of COVID-19, this concept relates to the large number of undiagnosed, asymptomatic carriers. Each individual cannot know the objective probability of being infected and thus must formulate his/her own guess, which is a subjective probability. In other words, there is uncertainty about the correctness of risks of infection. This implies that people face severe uncertainty about the expected amount of public goods provided by others, which in turn will impact their own social distancing behaviors. Previous research demonstrates that people tend to have ‘ambiguity aversion’ [21]. The presence of ambiguity makes people uncomfortable, and most make their decision based on a pessimistic scenario [22,23]. Existing theoretical works suggest that ambiguity aversion tends to enhance the provision of public goods; that is, free-rider incentives are suppressed [24,25]. This implies that ambiguity due to infodemics from social media can mitigate the free-rider problem under the COVID-19 pandemic. This is consistent with theoretical expectations established in a single person framework [12].

In sum, the literature on public goods suggests that infodemics, as sources of risk and ambiguity, can enhance social distancing, contrary to warnings from international organizations. This potential positive effect is consistent with recent studies on pandemic behavior in Japan. For example, individuals who frequently use Twitter, Facebook, and Instagram engage in more social distancing, but there is no effect from accessing traditional mass media, such as television of newspapers [13]. The theoretical mechanism posited in this paper, however, should apply to both social and mass medias. To test our posited framework, we use data from an original, multi-wave online panel survey in Japan. Our data covers two time periods: the very early stage of the pandemic (April 28^th^ to May 7^th^, 2020; n = 2,167), and the second wave of the pandemic (September 3^rd^ to 9^th^; n = 1662) when the same respondents were re-surveyed. The difference in sample sizes is due to respondent attrition, roughly 23%. Respondents in both waves were restricted to those in their 30s and 40s. They were recruited using quota sampling with respect to gender (two categories), age group (four 5-year categories), and location of residence (10 categories), so that their demographic distribution matched the latest census. The online Appendix includes the summary statistics.

Social distancing behavior is measured by whether the respondent reduced his/her reported frequency of dining out (1 if yes, 0 if no). We use a logistic regression model, with the key explanatory factors being media exposure. We include covariates for demographic traits, including educational attainment, gender, and age, as well as prefecture (region) fixed effects. Six types of media, defined broadly, are considered in this study. LINE, a WhatsApp-like service for sending text messages and video calls, is the most popular social media platform; the number of LINE users is 88 million, which corresponds to 69% of the entire population, according to ‘LINE Business Guide: June-December’ (cf. the online Appendix). Over 60% of respondents are daily users of LINE, while only 25% are daily users of Twitter. We also consider web-based news aggregators/curation platforms, which collect various pieces of information from online news, Wikipedia, Twitter, and other social media without confirming their correctness; 58% of respondents are daily users of these news aggregators (for example, ‘NAVER MATOME’ was one of the most commonly used web aggregators in Japan; it was terminated in September 2020, reportedly because its contents violated copyright laws). We also consider several mass media outlets: (a) TV (82%), (b) online news sites (34%), and (c) print newspapers (28%). Among the population in Japan, information acquisition from the internet has become quite common, although classic mass media remains popular; over 60% of people think that TV is trustworthy [26]. Analyses of infodemics in China have emphasized the major role of TV and WeChat, a WhatsApp-like service in China, as major sources of COVID-19 information [3]. This is consistent with the fraction of daily users of TV and LINE among our respondents (cf. Table A.1 in the online Appendix).

Table 1 shows that social media infodemics may increase social distancing behavior during the early stage of the pandemic. First, those who use certain types of social media on a daily basis are more likely to engage in protective, social distancing behaviors. Table 1 shows that daily users of LINE follow higher standards of social distancing. A similar pattern is found for daily users of web aggregators and television news. Notably, TV, LINE, and web aggregators are widely accessed by our survey respondents. This suggests that major media platforms – whether social or mass – may be sources of the mitigation mechanism. By contrast, Twitter users are neither more nor less likely to follow social-distancing measures than non-users. Table 2 shows that very similar patterns are found during the second wave of the pandemic. We note that ambiguity was substantially reduced in September compared to April, because there was more objective/correct information about COVID as time elapsed. Thus, our observation from the two tables suggests that ambiguity does not have a significant effect on social distancing in these periods; if it had, then the coefficients in Table 2 should be substantively and statistically less significant than in Table 1. This is not inconsistent with existing experimental works on ambiguity [12].

**Table 1.** Reducing the frequency of dining out in April-May and media usage: results on Logistic regression

	(1)	(2)	(3)	(4)	(5)	(6)
Daily LINE user	2.03***					
	(1.73, 2.39)					
	[<.001]					
Daily Twitter user		0.86				
		(0.72, 1.03)				
		[.107]				
Daily web aggregator user			1.56***			
			(1.28, 1.90)			
			[<.001]			
Daily TV user				1.52***		
				(1.28, 1.80)		
				[<.001]		
Daily online news user					1.39***	
					(1.18, 1.63)	
					[<.001]	
Daily newspaper user						0.97
						(0.75, 1.24)
						[.786]
University	1.25*	1.25*	1.26*	1.23*	1.22*	1.25*
	(1.02, 1.54)	(1.02, 1.54)	(1.02, 1.55)	(1.01, 1.51)	(1.00, 1.49)	(1.02, 1.54)
	[.035]	[.030]	[.029]	[.044]	[.048]	[.030]
Female	1.39**	1.53***	1.56***	1.48***	1.60***	1.53***
	(1.12, 1.74)	(1.24, 1.89)	(1.25, 1.95)	(1.19, 1.86)	(1.29, 1.98)	(1.24, 1.88)
	[.004]	[<.001]	[<.001]	[.001]	[<.001]	[<.001]
Age	0.99*	0.97***	0.97***	0.97***	0.97***	0.97***
	(0.97, 1.00)	(0.96, 0.99)	(0.96, 0.99)	(0.96, 0.99)	(0.96, 0.99)	(0.96, 0.99)
	[.045]	[<.001]	[<.001]	[<.001]	[<.001]	[<.001]
Prefecture FE	YES	YES	YES	YES	YES	YES
n	2,167	2,167	2,167	2,167	2,167	2,167
Hosmer-Lemeshow p-value	.406	.551	.052	.419	.828	.582

The dependent variable is the answer to the following question: ‘has your frequency of going out for dinners increased or decreased since last March?.’ All the specifications control for prefecture (region) fixed effects. The odds ratios are reported. Standard errors are clustered at the prefecture level. 95% CI are in parentheses. p-values are in brackets. *** p < 0.001, ** p < 0.01, * p < 0.05.

**Table 2.** Reducing the frequency of dining out in September and media usage: results on Logistic regression

	(1)	(2)	(3)	(4)	(5)	(6)
Daily LINE user	1.53***					
	(1.24, 1.88)					
	[<.001]					
Daily Twitter user		1.00				
		(0.77, 1.28)				
		[.972]				
Daily web aggregator user			1.22*			
			(1.01, 1.47)			
			[.040]			
Daily TV user				1.43*		
				(1.07, 1.91)		
				[.015]		
Daily online news user					1.37*	
					(1.06, 1.77)	
					[.018]	
Daily newspaper user						1.05
						(0.85, 1.29)
						[.665]
University	0.90	0.91	0.91	0.89	0.88	0.90
	(0.75, 1.09)	(0.75, 1.10)	(0.75, 1.10)	(0.74, 1.08)	(0.72, 1.08)	(0.74, 1.10)
	[.288]	[.319]	[.329]	[.249]	[.229]	[.313]
Female	1.18*	1.25**	1.25**	1.21*	1.29**	1.25**
	(1.01, 1.39)	(1.06, 1.47)	(1.07, 1.47)	(1.02, 1.43)	(1.10, 1.50)	(1.06, 1.48)
	[.042]	[.008]	[.006]	[.027]	[.001]	[.009]
Age	1.02	1.01	1.01	1.01	1.01	1.01
	(1.00, 1.04)	(0.99, 1.03)	(0.99, 1.03)	(0.99, 1.03)	(0.99, 1.03)	(0.99, 1.03)
	[.065]	[.189]	[.198]	[.217]	[.223]	[.217]
Prefecture FE	YES	YES	YES	YES	YES	YES
n	1,662	1,662	1,662	1,662	1,662	1,662
Hosmer-Lemeshow p-value	.253	.967	.946	.782	.787	.930

The dependent variable is the answer to the following question: ‘has your frequency of going out for dinners increased or decreased since last March?.’ It was elicited in the resurvey, and therefore, the sample size is smaller than Table 1 due to the attrition. All the specifications control for prefecture (region) fixed effects. The odds ratios are reported. Standard errors are clustered at the prefecture level. 95% CI are in parentheses. p-values are in brackets. *** p < 0.001, ** p < 0.01, * p < 0.05.

## Public health implications

Let us conclude with some public health implications. The first is straightforward: although governments have sought to contain media-generated COVID-19-related infodemics, these may actually have positive effects in encouraging social distancing, a public good. Therefore, it may not necessarily be socially desirable to suppress all pieces of misinformation or misunderstanding, as both traditional/mass and new/social medias can work effectively as a mitigation device. In the case of novel diseases, even governments and experts may not know the correctness of information perfectly. Accordingly, one reasonable response may be to remain neutral on infodemics regarding ‘unharmful’ protective behaviors with limited negative consequences, such as wearing masks, eating healthy foods, or taking off shoes inside a house.

However, even if infodemics have a mitigation effect on the insufficiency of social distancing, there are several reasons why governments may want to be wary of infodemics as dangers to public health. First, the impact of infodemics on individuals’ risk perception is heterogeneous. Optimistic individuals who are exposed to positive information may be less likely to follow social distancing guidelines. Even if infodemics increase social distancing on average, it may decrease protective behavior among a minority of people. While this share may be tiny, it can still produce clusters of infection with dire social consequences. Put differently, the heterogeneity in beliefs caused by infodemics can be a potential danger for infection control. Such heterogeneity may also cause a disparity in preferences over policies toward COVID-19, including the necessity of stronger measures such as shelter-at-home orders and business closures. As a result, the political and social costs of conflicts over policies can be very high. Second, some types of misinformation have serious negative effects [27,28]. For example, COVID-19 vaccine misinformation has caused a large number of people to postpone or avoid vaccination, posing fatal risks to their wellbeing.

We end with some limitations of our study. In our theoretical argument, we consider only a simple extension of the public-good model with rational agents. However, under the pandemic, there are various socio-psychological factors that our model cannot capture. First, people are not always rational and their behaviors can be biased. Second, although our theory is static, a dynamic model is important in the context of epidemiology [29]. Third, in our empirical analysis, we do not have variables that can be used to identify a difference between the effects of risk and ambiguity. For these matters, we hope (and urge others) to conduct further research.

## Paper context

This study examines the impact of infodemics—an over-abundance of information—on social distancing under the COVID-19 pandemic. Although the negative effects of infodemics have been emphasized by governments and international organizations, theoretical models suggest that infodemics may work as a mitigation mechanism. Utilizing empirical evidence of this mitigation effect based on an original survey, we argue that an over-abundance of information can actually encourage social distancing. However battling infodemics requires a long-run perspective that takes mental health into account.

## References

[1] Maragakis LL. Coronavirus, social and physical distancing and self-quarantine. Johns Hopkins Medicine; 2020 [cited 2020 Jun 23]. Available from: https://www.hopkinsmedicine.org/health/conditions-and-diseases/coronavirus/coronavirus-social-distancing-and-self-quarantine

[2] Cato S, Iida T, Ishida K, et al. Social distancing as a public good under the COVID-19 pandemic. Public Health. 2020 Nov;188:51–6. 33120232 10.1016/j.puhe.2020.08.005 PMC7425541

[3] Zarocostas J. How to fight an infodemic. Lancet. 2020 Feb;395(10225):676. 32113495 10.1016/S0140-6736(20)30461-X PMC7133615

[4] Hua J, Shaw R. Corona virus (Covid-19) “infodemic” and emerging issues through a data lens: the case of China. Int J Environ Res Public Health. 2020 Mar;17(7):2309. 10.3390/ijerph17072309 PMC7177854 32235433

[5] Naeem SB, Bhatti R. The Covid‐19 ‘infodemic’: a new front for information professionals. Health Info Libr J. 2020 Sep;37(3):233–239. 32533803 10.1111/hir.12311 PMC7323420

[6] WHO Coronavirus disease 2019. (COVID-19) situation Report – 86; 2020 [cited 2020 Jun 23]. Available from: https://www.who.int/emergencies/diseases/novel-coronavirus-2019/situation-reports

[7] Li HO, Bailey A, Huynh D, et al. YouTube as a source of information on COVID-19: a pandemic of misinformation? BMJ Glob Health. 2020 Apr;5(5):e002604. 10.1136/bmjgh-2020-002604 PMC7228483 32409327

[8] Mourad A, Srour A, Harmanani H, et al. Critical impact of social networks infodemic on defeating coronavirus COVID-19 pandemic: twitter-based study and research directions. arXiv preprint arXiv:2005.08820. 2020.

[9] Brodeur A, Grigoryeva I, Kattan L. Stay-at-home orders, social distancing, and trust. J Popul Econ. 2021 Aug;34(4):1321–1354. 10.1007/s00148-021-00848-z PMC8214058 34177123

[10] Muto K, Yamamoto I, Nagasu M, et al. Japanese citizens’ behavioral changes and preparedness against COVID-19: an online survey during the early phase of the pandemic. PloS One. 2020 Jun;15(6):e0234292. 32525881 10.1371/journal.pone.0234292 PMC7289361

[11] Shoji M, Cato S, Iida T, et al. and social-distancing in the absence of legal enforcement: survey evidence from Japan. MPRA Paper No. 101968. 2020.

[12] Kishishita D, Tung H, Wang C. Ambiguity and self-protection: evidence from social distancing under the COVID-19 Pandemic. Available at SSRN 3778645. 2021. 10.1007/s42973-022-00120-3 PMC9525947 36213493

[13] Cato S, Iida T, Ishida K, et al. The bright and dark sides of social media usage during the COVID-19 pandemic: survey evidence from Japan. Int J Disaster Risk Reduct. 2021 Feb;54:102034. 10.1016/j.ijdrr.2020.102034 PMC9766757 36570032

[14] Samuelson PA. The pure theory of public expenditure. Rev Econ Stat. 1954 Nov;36(4):387–389.

[15] Bergstrom T, Blume L, Varian H. On the private provision of public goods. J Public Econ. 1986 Feb;29(1):25–49.

[16] Olson M. The logic of collective action: public goods and the theory of group. Cambridge MA: Harvard University Press; 1965.

[17] Ostrom E. Collective action and the evolution of social norms. J Econ Perspect. 2000 Summer;14(3):137–158.

[18] Anomaly J. Public health and public goods. Public Health Ethics. 2011 Nov;4(3):251–259.

[19] Dees RH. Public health and normative public goods. Public Health Ethics. 2018 Apr;11(1):20–26.

[20] Austen-Smith D. Individual contribution to public goods. Econ Lett. 1980 Sep;5(4):359–361.

[21] Ellsberg D. Risk, ambiguity, and the Savage axioms. Q J Econ. 1961 Nov;75(4):643–669.

[22] Viscusi WK, Chesson H. Hopes and fears: the conflicting effects of risk ambiguity. Theory Decis. 1999 Oct;47(2):157–184.

[23] Etner J, Jeleva M, Tallon JM. Decision theory under ambiguity. J Econ Surv. 2012 Apr;26(2):234–270.

[24] Eichberger J, Kelsey D. Strategic complements, substitutes, and ambiguity: the implications for public goods. J Econ Theory. 2002 Feb;106(2):436–466.

[25] Bailey RW, Eichberger J, Kelsey D. Ambiguity and public good provision in large societies. J Public Econ Theory. 2005 Dec;7(5):741–759.

[26] Joho-Tsushin-Hakusyo. Ministry of Internal Affairs and Communications of Japan; 2019. Available from: https://www.soumu.go.jp/johotsusintokei/whitepaper/ja/r01/html/nd114120.html

[27] Germani F, Biller-Andorno N, Lavorgna L. The anti-vaccination infodemic on social media: a behavioral analysis. PloS One. 2021 Mar;16(3):e0247642. 33657152 10.1371/journal.pone.0247642 PMC7928468

[28] Loomba S, de Figueiredo A, Piatek SJ, et al. Measuring the impact of COVID-19 vaccine misinformation on vaccination intent in the UK and USA. Nat Hum Behav. 2021 Feb;5(3):337–348. 33547453 10.1038/s41562-021-01056-1

[29] Fenichel EP. Ecoomic considerations for social distancing and behavioral based policies during an epidemic. J Health Econ. 2013 Mar;32(2):440–451. 23419635 10.1016/j.jhealeco.2013.01.002 PMC3659402

